# Genetically Modified Micro-Organisms for Industrial Food Enzyme Production: An Overview

**DOI:** 10.3390/foods9030326

**Published:** 2020-03-11

**Authors:** Marie Deckers, Dieter Deforce, Marie-Alice Fraiture, Nancy H.C. Roosens

**Affiliations:** 1 Transversal activities in Applied Genomics (TAG), Sciensano, Rue Juliette Wytsmanstraat 14, 1050 Brussels, Belgium; marie.deckers@ugent.be (M.D.); marie-alice.fraiture@sciensano.be (M.-A.F.); 2 Laboratory of Pharmaceutical Biotechnology, Ghent University, Campus Heymans, Ottergemsesteenweg 460, B-9000 Ghent, Belgium; dieter.deforce@ugent.be

**Keywords:** food enzymes, genetically modified micro-organisms, European regulations, safety control

## Abstract

The use of food enzymes (FE) by the industrial food industry is continuously increasing. These FE are mainly obtained by microbial fermentation, for which both wild-type (WT) and genetically modified (GM) strains are used. The FE production yield can be increased by optimizing the fermentation process, either by using genetically modified micro-organism (GMM) strains or by producing recombinant enzymes. This review provides a general overview of the different methods used to produce FE preparations and how the use of GMM can increase the production yield. Additionally, information regarding the construction of these GMM strains is provided. Thereafter, an overview of the different European regulations concerning the authorization of FE preparations on the European market and the use of GMM strains is given. Potential issues related to the authorization and control of FE preparations sold on the European market are then identified and illustrated by a case study. This process highlighted the importance for control of FE preparations and the consequent need for appropriate detection methods targeting the presence of GMM, which is used in fermentation products.

## 1. Introduction

Enzymes can be found in all living organisms and are proteins that catalyze biochemical reactions. For decades, enzymes have been used in food processes, such as for the clarification and filtering of wines and beers, for baking, for the production of cheeses, and much more [1].

Industrially, enzymes are mostly used in the paper and pulp industry, the textile industry, the pharmaceutical industry, and the food industry. In 2016, the market value of industrial enzymes was estimated at USD 4.61 billion, with an expected growth of 5.8% between 2017 and 2022, reaching an estimated value of USD 6.3 billion [2,3]. The use of enzymes for food and beverage manufacturing in particular represents a big part of the industrial enzyme market, which is expected to reach a value of USD 2.3 billion by 2021 [4,5].

In order to be used by the industry, enzymes can either be extracted from plant and animal sources or they can be obtained through fermentation using bacterial or fungal strains, for which both wild-type (WT) or genetically modified (GM) strains, which have been optimized to increase the protein production and limit the production of unwanted secondary metabolites, can be used [6].

Within the European Union (EU), multiple regulations ((EC) No. 1331/2008, (EC) No. 1332/2008, (EC) No. 1333/2008, and (EC) No. 1334/2008) have been published to harmonize the evaluation of food enzymes (FE), additives, and flavorings [7,8,9]. These regulations require a safety evaluation of all FE, additives, and flavorings before they are allowed to be sold on the EU market. Therefore, FE applicants are requested to submit dossiers for all FE that are and will be sold on the EU market. One of the key criteria of the safety evaluations is the absence of the production strain (whether genetically modified or not) in the final FE preparations. As a result of these regulations, the need for an appropriate control by enforcement laboratories of commercialized FE preparations is growing.

In this review, a general overview will be provided of the industrial production of FE preparations, with a particular focus on the use of genetically modified micro-organisms (GMM).

Firstly, after describing the industrial enzyme production process, an outline of the characteristics of GMM used during the FE fermentation process will be provided. This will mainly include a description of the selection criteria to choose an appropriate host micro-organism and the selection of appropriate vectors and selection markers. Secondly, the various European FE regulations and the consequent safety evaluations of FE preparations will be described. Thirdly, an overview of the currently submitted FE dossiers and the key information that could be extracted from these dossiers will be represented. Fourthly, a resume of the different GMO regulations and their applicability to FE preparations will be provided. Lastly, a case study will be described, representing the analysis of a commercialized FE preparation, resulting in the detection of an unauthorized GMM and highlighting the importance of developing appropriate workflows for the control and monitoring of FE preparations.

## 2. Industrial Production of Food Enzymes

### 2.1. Fermentation Processes

Food enzymes (FE) can either be obtained from micro-organisms (MO), by fermentation, or from animal and plant sources by extraction. Previously, the use of enzymes originating from plant and animal sources was preferred, because these enzymes were deemed to be free of contaminants that are often associated with microbial (MB) fermentations. However, over the years, the advantages of MB fermentations could not be neglected, such as their cost efficiency, technical, and ethical advantages (regarding animal sources) [10].

For the production of enzymes by MO, different types of fermentation processes can be used, such as submerged fermentation (SmF) and solid-state fermentation (SSF) [6].

SmF uses an excess of free water and high oxygen concentrations to nurture the MO and requires the use of stirrers and impellers to mix all the components. This production process is widely used by the food industry, thanks to the ease of handling at large scale and possibilities for online control of several parameters, such as temperature and pH. Four different types of SmF exist, depending on the culturing method of the MO in the fermenter. These are batch, fed-batch, perfusion, or continuous culture methods [11].

If a fungal strain is used for the enzyme production, a viscous solution can be obtained using SmF as the mycelium grows, which can hinder the impellers [6]. Therefore, SSF are often preferred for fungal fermentations. Additionally, downstream processing for SSF is often less expensive than for SmF. Other advantages are the high productivity, higher product concentration, and more simple fermentation equipment thanks to lower effluent generation. During SSF, the MO grow on a solid substrate, wherein the necessary substrates for growth and enhancement of enzyme production are present; however, if needed, additional substrates can be supplemented in this solid phase.

### 2.2. Purification

Following the fermentation process, downstream processing is necessary to obtain purified enzyme concentrates. Depending on the type of fermentation used, whether the enzyme is produced intracellularly or extracellularly, and depending on the end use of the enzyme, different purification steps are used. For instance, enzymes with pharmaceutical uses will go through a stricter purification process than enzymes used as raw materials in feed [1].

For enzymes produced intracellularly, the cell walls and membranes need to be disrupted first, either by mechanical or enzymatic methods. However, in most cases, the enzymes are produced extracellularly, where the first purification step consists of the separation of the cells from the fermentation broth. For separation of fungal cells, centrifugation can be used at low speed. Bacterial cells can be separated by flocculation, for instance with aluminum sulphate, which will negate the charge of the bacterial membrane and result in clumps that will then be excluded from the suspension. Thereafter, multiple steps based on filtration, centrifugation, flocculation, crystallization, electrophoresis, and chromatography are used to obtain the desired purification level of the enzyme concentrate [11].

### 2.3. Immobilization

During food production processes, both soluble enzymes and immobilized enzymes can be used. Traditionally, soluble enzymes have been used in batch processes, with the use of stirred tank reactors. In this case, the product needs to be separated from unused substrates and the enzyme at the end of the process. However, these separation processes are costly and often cause a denaturation of the enzyme, and therefore require the use of new enzymes for the next batch run [12].

Immobilized enzymes allow the reuse of enzymes for multiple batches, and therefore reduce the total cost of the needed enzymes. Immobilization can be based on adsorption, covalent bonding, or entrapment of the enzyme on or in supporting matrices [13]. The use of immobilized enzymes allows the food producer to use continuous-flow reactors with a continuous supply of substrates and with extraction of products. Even though the immobilized enzymes are less effective, such continuous reactors have lower operational costs than batch processes, thanks to having higher process efficiency, less product variation, and smaller production facilities. Therefore they are very suitable for industrial-scale production [1,13,14].

### 2.4. Formulation

Enzymes can be found under three different forms. Firstly, there is the enzyme protein itself, which is a pure substance that is used in laboratories. Secondly, enzyme concentrates are the products obtained after fermentation or extraction. They contain the produced enzyme together with much smaller quantities of other substances obtained during the fermentation process, such as other (secondary) enzymes or remaining products from the fermentation. Those enzyme concentrates are subject to a safety evaluation before their approval for commercialization. Lastly, there are enzyme preparations, which are formulations containing one or multiple enzyme concentrates with the addition of stabilizers, preservatives, and diluents to stabilize the enzymes and allow conservation of the activity. These formulations are what are sold on the market [8,15,16].

Both liquid and solid preparations are possible. However, if possible, liquid preparations are preferred to solid preparations due the tendency of solid preparations to form dust and consequently sometimes cause allergic respiratory reactions [11].

## 3. GMM Used for Optimization of Food Enzyme Production

### 3.1. Advantages of Using Micro-Organisms to Produce FE

Industrial enzymes are produced for 50% by fungi, 35% by bacteria, and 15% by animal and plant sources [17,18]. MB sources are preferred for the production of enzymes (mainly for economic and technical reasons) in comparison with extraction from plant and animal sources [1,19]. The advantages can be summarized as follows: (I) Many MO produce enzymes extracellularly, which simplifies the extraction method during the purification. Even when the production of the enzyme is intracellular, the disruption of MB cells is easier than the extraction from plants and animals [1]. (II) Particular MB strains can be selected to obtain well-characterized enzymes with specific properties [20]. (III) Due to a shorter production time, smaller production facilities are needed, resulting in a reduced production cost. Additionally, animals and plants need to be transported to extraction facilities, which are not needed for MB fermentations, where the whole production takes place in only one location [19]. (IV) Higher yields can be obtained for MB enzymes. Moreover, the availability of enzymes from animal and plant sources is also depended on the time of the year, therefore reducing the total yield and limiting the ability for continuous production [5]. (V) MB enzymes usually have a higher activity and stability. For instance, enzymes obtained from thermophilic MO will have a higher temperature tolerance [21]. (VI) MO can easily be GM in order to obtain optimized enzyme products, higher yields, and better characteristics. Additionally, modifications of animals and plants are technically more difficult, and modifications of animals, especially, are ethically more sensitive [6].

Considering the different advantages of using MO for FE production, the ease of introducing genetic modifications in the production MB strains to further improve the industrial enzyme production is probably one of the biggest advantages. These modifications can be obtained by either the genetic modification of the production strain or by the use of recombinant enzymes. The use of GMM for the production of food enzymes allows the production yield to be increased, improving the characteristics of the produced enzyme, limiting the production of unwanted metabolites (such as mycotoxins), and expressing enzymes in organisms that normally would not produce this enzyme [22]. Recombinant enzymes are used to improve an enzyme’s characteristics, such as its activity, temperature optimum, and pH stability [23].

### 3.2. Characteristics of Genetically Modified Micro-Organsisms

The use of GMM for industrial production of FE is advantageous because it allows the combination of appropriate production sources with the production of desired enzymes. For example, in the case of a strain producing a native enzyme that is not suitable for industrial applications, which is the case for pathogenic strains, the enzyme can be produced by another organism that is more suitable in terms of food safety. Genetic modifications are also used if unwanted secondary metabolites are produced, such as toxins, where native genes can be deleted to hinder their expression [19].

In order to construct a successful recombinant production strain, several points need to be taken into account. First, it is important to select a host organism containing the appropriate characteristics for the production and expression of the desired enzyme and that is relatively cheap to culture. Then, the type of expression vectors, the employed transformation methods, and the selection markers used to select the transformed strains need to be determined, which are all important parameters for obtaining an optimal GMM for industrial use [22,24].

#### 3.2.1. Selection of Host Organism

Bacteria are simple and cheap host organisms, with many expression systems available for recombination, resulting in easy integration of modifications. However, due to issues related to protein folding, only simple proteins can be produced. Additionally, bacteria are not able to perform post-translational modifications. In contrast, fungal host strains contain a subcellular organization, allowing correct folding of the proteins and post-translational modifications. Additionally, fungi secrete only a limited amount of their own secondary proteins into the medium, facilitating the subsequent purification steps [18]. On the other hand, the production of non-fungal recombinant proteins is generally low in filamentous fungi [22,25]. Therefore, the selection of the appropriate host strain is on a case-by-case analysis.

Bacterial host strains that are commonly used are *Escherichia coli*, *Bacillus subtilis*, *Bacillus licheniformis*, and *Bacillus amyloliquefaciens*. For *E. coli*, many molecular toolboxes are available, facilitating the construction of expression systems suitable for high-yield enzyme production. However, the proteins are expressed in the cytoplasm, resulting in the need for additional purification steps. The various *Bacillus* species, however, can secrete higher concentrations of the enzymes into the medium [23]. Therefore, *B. subtilis*, for example, is mainly used for the production of α-amylase and proteases [26].

Commonly used filamentous fungi are *Aspergillus niger, Aspergillus oryzae*, and *Trichoderma reesei* [27]. *A. oryzae*, for example, has been used for decades for the production of fermented food, but it is also known to produce the mycotoxin aflatoxin. To circumvent this issue, the mutant strain BECh2 was constructed, in which the aflatoxin gene cluster and the gene-synthesizing cyclopiazonic acid were deleted and kojic acid production was reduced [19,22]. This mutant strain is currently used as a host strain for the production of triacylglycerol lipase and glucose oxidase, among others [28,29].

#### 3.2.2. Expression Vectors and Transformation Processes

**Figure 1:**
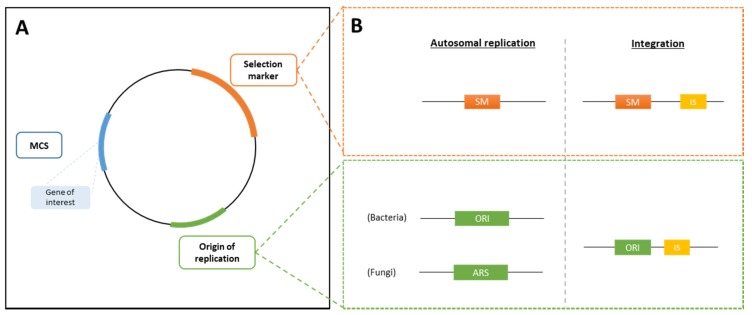
Schematic representation of an expression vector. (**A**) Expression vectors contain an origin of replication (ORI), followed by the multiple cloning site (MCS) where the gene of interest is integrated and a selection marker (SM). (**B**) Both an episomal plasmid and integrative plasmid, containing flanked integration sites, can be created. ARS, autonomous replication sequence; IS, integration site.

Expression vectors are used for the integration of modifications into the selected host strain. An expression vector typically contains, at the least, an origin of replication (ORI), a multiple cloning site (MCS), in which the gene of interest will be integrated, and a selection marker (see Section 3.2.3) for selection of the recombinant strain (Figure 1A). Additionally, promoter and terminator regions are present to control the expression of the gene of interest and the selection marker (see Section 3.2.4). The size of the vector is kept as small as possible to avoid an increase of the metabolic load on the host organism and to avoid reduced plasmid stability [25].

Bacterial expression vectors can either be integrated into the host genome (integrative plasmid) or they can be extrachromosomal, using autonomously replicating plasmids (episomal vector). Yeast and fungal expression vectors are preferably integrated into the host genome. Additionally, for eukaryotes, shuttle vectors are commonly used, meaning that these vectors can be inserted into both prokaryotes and eukaryotes, and that they contain selection markers that work in both MB groups. Figure 1A and Figure 1B show a simple representation of the different necessary parts of an expression vector, for both an episomal vector and for an integrative plasmid.

For insertions into the host genome, the integrative plasmid contains two integration sites (IS) flanking the MCS, which allow the cleavage of the vector and subsequent integration by recombination into the host genome containing homologous sequences to the IS. The integration is usually performed into a specific locus. In this case, an extension needs to be added to the expression cassette, which is homologous to a specific targeted site [30].

Episomal plasmids require the presence of an origin of replication (ORI) that is compatible with that of the host strain. For autonomous replication in eukaryotes, an autonomous replication sequence (ARS) is needed, which is similar to the ORI found in prokaryotes [31].

Various transformation methods can be used to transfer the vector DNA into the host organism. For bacterial host strains, approaches include conjugation (based on cell-to-cell contact), electroporation, the use of competent cells, or vector incubation using protoplasts. This latter method is generally used for the transformation of eukaryotes [22].

#### 3.2.3. Selection Markers

As not all targeted strains are successfully transformed, it is important to include a selection step to retain only the recombinant strains for further purposes.

For prokaryotes and some eukaryotes, the selection step is mainly based on the integration of antimicrobial resistance (AMR) genes. The used selection marker can either be specific for eukaryotes or prokaryotes or it can be applicable to both at the same time [32]. Alternatively, auxotrophic mutants can be used in order to avoid the use of AMR markers that are associated with public health concerns. In this later case, a specific amino acid or other nutrient is needed for the growth of the modified host strain [22]. This is preferred for industrial applications when a selection pressure needs to be maintained during the whole production, in order to guaranty the stability of the introduced plasmid [25].

Most bacterial expression vectors contain kanamycin (kanR), ampicillin (AmpR), and tetracycline (TetR) resistance genes [22]. Selection markers used for filamentous fungi are generally auxotrophic markers [19]. One such example is the amdS marker, which encodes for acetamidase, hydrolyzing acetamide into acetate and ammonia. Strains that have been transformed with amdS can be selected by using acetamide as the sole nitrogen source [22]. This selection marker is commonly chosen because the often-used host strains *A. niger* and *A. oryzae* lack an endogenous *amdS* gene. This amdS gene was used as a dominant marker in an industrial *A. niger* strain from DSM [30]. Another often-used auxotrophic selection marker is the *URA3* gene from *Saccharomyces cerevisiae*, encoding orotidine 5′-monophosphate, which is an essential enzyme in the pyrimidine biosynthesis [22,33].

#### 3.2.4. Increased Gene Expression

In order to obtain a high enzyme yield, an efficient gene expression of the gene of interest is also important. One of the most commonly used strategies to increase the expression yield is performed through multiple integration [34]. To this end, various strategies can be used, such as: (i) using high amounts of DNA during the transformation; (ii) applying a strong antibiotic pressure, which can be done by using a high antibiotic concentration or by using a weak promoter for the selection marker; or (iii) by simultaneously or consecutively integrating multiple expression constructs containing different selection markers or by using two copies of the gene of interest in bi-directional expression vectors [25]. However, incorporation of multiple copies can result in stability issues in the obtained strain, which can be problematic in industrial-scale production, where a long-term cultivation procedure is required. An example of a strategy using antibiotic pressure to select strains containing multiple insertions is the post-transformational vector amplification (PTVA) method, which uses increasing antibiotic concentrations [35].

Another important factor to increase the gene expression is the strength of the promoter. The expression is often regulated by a set of activators and repressors that differentiate constitutive promoters from tunable promoters. Constitutive promoters are expressed independently of the environmentally induced transcription factors and regulate the expression of basal genes. In contrast, tunable promoters react to the presence or absence of biotic or abiotic factors that can induce or repress the promoter [36]. In *Bacillus* species, the *amyL* (*B. licheniformis* α-amylase) and *amyM* (*B. stearothermophilus* maltogenic amylase) genes are often used as promoters [22]. Regarding filamentous fungi, in *A. oryzae* the TAKA amylase promoter is often used [37]. This tunable promoter is induced by the transcription activator *amyR* (amylolytic gene expression) and is repressed by the regulator creA (carbon catabolite repressor) [19]. Another commonly used inducible promoter for *T. reesei* or other fungi, is the cellobiohydrolase I gene (cbh1) promoter, which is cellulose-induced [38,39].

A simple solution to the tandem repeat of mono-directional expression cassettes is the use of bi-directional promoters (BDPs). These primers allow a bi-directional gene expression of one or more genes [40]. Combined expression of both the gene of interest and a selection marker is also possible [41]. However, in order to allow the industrial use of these BDPs, larger sets of promoters must be available. Promoters representing variable expression ratios and combining different regulatory profiles per expression direction are needed. Therefore, a library of BDPs was established by [42], which in this particular case are to be used in the yeast *Pichia pastoris*, an emerging host organism.

### 3.3. New Technologies to Introduce Genetic Modifications

In recent years, new technologies have emerged for genetic engineering, such as CRISPR/Cas9. This stands for “Clustered Regularly Interspaced Short Palindromic Repeat” and “CRISPR-associated protein 9”, which originates from archaeal and bacterial immune systems [43]. The Cas9 endonuclease introduces double-stranded breaks (DSBs) into the target DNA. Using endogenous repair pathways, the genomic DNA can either be altered by introducing mutations or by introducing a specific donor sequence. This is mediated by the non-homologous end-joining (NHEJ) DNA repair mechanism or by the homologous repair (HR) system [43,44]. In contrast to eukaryotes, the NHEJ DNA repair system is absent in most bacteria. Therefore, these bacteria are unable to repair the CRISPR/Cas9-mediated breaks, resulting in the death of wild-type strains [45]. This allows the CRISPR/Cas9 system to be used for selection of recombinant strains and as an alternative to antimicrobial resistance (AMR) genes [44].

Using this technique, the extracellular pullulanase production were enhanced in the *B. subtilis* strain WS5. Since proteases can degrade heterologous enzymes, such as for pullulanase, genes related to the protease production were disrupted [46]. Genes related to unwanted characteristics, such as foam production during the fermentation or increased spore formation, can also be disrupted to produce a more useful strain for industrial enzyme production [47]. CRISPR/Cas has also been used for the modification of eukaryotes. For example, an industrial *Penicillium subrubescens* strain has been developed. In this particular example, the *ku70* gene was deleted, which is involved in the non-homologous end-joining (NHEJ) DNA repair mechanism. With this deletion, the homologous repair (HR) system is favored, and therefore the insertion of a specific and desired DNA sequence into the cleavage site is favored [48].

### 3.4. Development of Recombinant Enzymes

**Figure 2:**
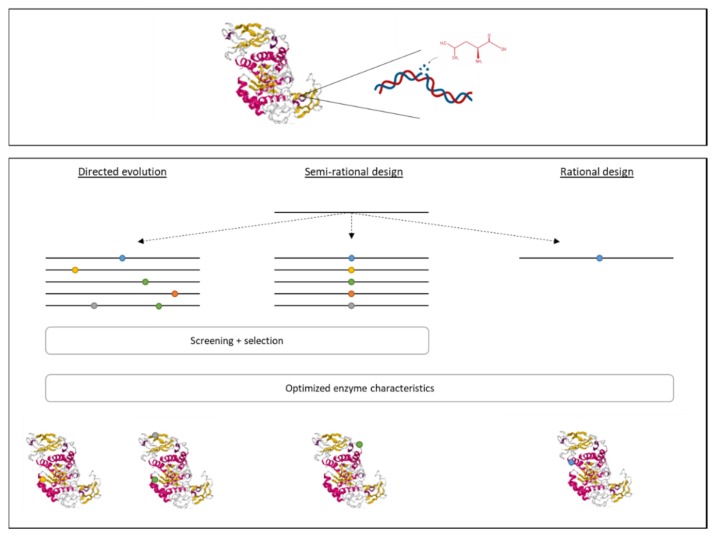
The construction of recombinant proteins can be done using three different approaches. Rational design is used to modify a specific amino acid that is known to be related to characteristics that need to be changed. If no information is known about the enzyme’s structure and mechanism, both directed evolution and semi-rational design can be used. For directed evolution, a library is prepared of random mutations, mimicking evaluation. This is followed by a thorough screening and selection process. The semi-rational design combines the knowledge of the enzyme’s structure to know where a change is needed with the methodology of directed evolution to prepare a library of mutations within a specific region.

In addition to genetic modifications of the producing strain, it is also possible to modify the enzyme itself in order to obtain an improved yield and improve the enzyme’s characteristics. There are multiple techniques that can be used to generate recombinant enzymes, such as rational design, directed evolution, and semi-rational design (Figure 2), depending on the amount of knowledge about the 3D structure and function of the enzyme, the enzyme’s sequence, and the modifications to be made to obtain the desired result [10,16,49].

(i) The rational design is based on the replacement, insertion, or deletion of a specific DNA region encoding for an amino acid related to a certain activity that is to be improved. Information regarding the 3D structure of the protein and its chemical mechanism are necessary. Usually, site-directed mutagenesis is used to replace a specific amino acid by another in order to obtain the desired functionality [10,23,49]. For example, a thermostable pullulanase from *Bacillus naganoensis* was obtained by rational design. This enzyme is used to hydrolyze amylopectin to amylose in starch [20].

(ii) Directed evolution consists of two steps. First, genetic diversity is created by random mutagenesis, mimicking evolution. This can be done by error-prone PCR, chemical mutagenesis, or UV radiation. Then, a thorough screening and evaluation process is needed, which is often agar-plate based, to select the modified enzymes that possess the desired characteristics. For this approach, it is not required to have knowledge about the enzyme’s structure and functions [10,49].

(iii) The semi-rational design combines the two previous approaches. In this case, information is available about the enzyme’s structure and functions. However, there is no knowledge about the modification to be made to obtain the desired result. Therefore, in the first step, amino acids are mutated within a specific region (related to the targeted characteristic). This is followed by a screening and evaluation step, such as for the directed evolution [10,23].

## 4. European Food Enzyme Regulations and Consequent Safety Evaluations: Current Status and Challenges

### 4.1. Food Additive or Processing Aid

FE can be categorized as a food additive or as a processing aid, depending on the effect it has on the food product. These two different categories result in different regulations regarding the labelling requirements.

A food additive is intentionally added to food to exert a technological function within the food. This can be done during the manufacture, processing, preparation, treatment, packaging, transport, or storage of the food. Food additives require a European authorization (following the regulation (EC) No 1129/2011 [50]). This includes a risk assessment performed by the European Food Safety Agency (EFSA).

A processing aid is a substance added to food to exert a technological function during the food processing, but not in the final food itself. Processing aids are currently still regulated at national levels in member states of the EU.

### 4.2. Overview of the Different European Regulations

Within the European Union (EU) and before 2008, the use of enzymes in the food industry was only regulated at the national level in France and Denmark, by the French “arretée” and the decree N 250 of 8 March 2013 [51,52], respectively. The industry itself was, and still is, responsible for the product quality of all enzymes sold in the EU market. In order to harmonize the regulations and subsequent safety evaluations of food additives, flavorings, and enzymes, the European Parliament published four regulations in 2008, being: (i) (EC) No. 1331/2008: Common Authorization Procedure; (ii) (EC) No. 1332/2008: Food Enzymes (iii); (EC) No. 1333/2008: Food Additives (iv); (EC) No. 1334/2008: Food Flavorings [7,8,9,10]. The aim of these four regulations is to obtain a harmonized safety evaluation of food additives, enzymes, and flavorings, resulting in three different community lists, respectively containing all food additives, enzymes, or flavorings allowed on the European market. In contrast to food additives and flavorings, no prior safety evaluations were performed for FE and no community list of authorized FE already existed. Therefore, according to (EC) No. 1332/2008 FE, producers and applicants were requested to submit FE dossiers to the EFSA (between 2011 and 2015) for evaluation prior to being added to a community list, which listed all authorized FE to be sold on the European market. The evaluation of FE consists of a risk assessment, performed by the EFSA and published as a scientific opinion, and a risk management based on this scientific opinion, performed by the European Commission (EC) and member states. These evaluations are currently being performed and 74 scientific opinions have currently been published.

Apart from the regulation (EC) No. 1332/2008, a few FE were already authorized for specific uses. According to the commission regulation (EU) No. 231/2012, laying down specifications for (EC) No. 1333/2008, both invertase (E 1103) and lysozyme (E 1105) are authorized for use as food additives [53]. Urease, β-glucanase, and lysozyme are also authorized for use in wine production according to regulation (EC) No. 1493/1999 [54]. All of these enzyme applications will be included in the community list, which will gather all authorized FE in one document, overriding prior authorizations, as mentioned in recital (16) from (EC) No. 1332/2008.

Multiple amendments and new regulations have been published since 2008. Regulation (EU) No. 234/2011 [55] implements regulation (EC) No. 1331/2008 and contains a list of the required information that needs to be integrated in the submission dossiers. It also contains the timeframe for submissions and evaluations. As an amendment to this regulation, (EU) No. 562/2012 [56] was published, allowing the submission of joint dossiers for companies submitting dossiers for the same FE. Consequently, The Association of Manufacturers and Formulators of Enzyme Products (AMFEP) has submitted 16 joint dossiers. As an addition to (EC) No. 1333/2008, regulation 2000/13/EC [57] was published to clarify the labelling requirements.

### 4.3. Qualified Presumption of Safety Status

In order to provide a harmonized safety evaluation of MO, the qualified presumption of safety (QPS) concept was introduced in 2003 [58]. This pre-assessment is used during the risk assessments performed by the EFSA. QPS status is granted after an intense literature search, based on the taxonomic identification, history of use, pathogenicity, and end use of the species. QPS status can also be granted to GMM strains if the recipient strain qualifies for the QPS status and the genetic modifications do not give rise to safety concerns.

In 2016, a first QPS list was published by EFSA [59]. Since then, a new QPS Panel Statement has been published every 6 months, containing evaluations of new notifications of MO for a possible QPS status, as well as a literature screening of all mentioned MO concerning possible new safety concerns. Additionally, every 3 years, a new QPS list is published in the QPS Opinion [60].

The QPS status can be compared to the Generally Recognized as Safe (GRAS) status given to safe MO by the U.S. Food and Drug Administration (FDA) [61].

### 4.4. Criteria and Challenges Regarding Microbial Presence in Food Enzyme Preparations

A key component of the safety evaluations performed by the EFSA of the FE preparations obtained from MB sources is the safety assessment of the production source itself, in particular the pathogenic and toxigenic potential. The process for the safety evaluation of microbial-derived food enzymes is schematically illustrated in Pariza and Johnson, 2001 [62]. Therefore, the applicant must provide information about the employed production source(s) to the EFSA in the submission dossiers. More details about the information that must be provided, including both technical data and toxicological data, can be found in regulation (EU) No. 234/2011 and in EFSA statements [63,64,65]. For example, the applicant must provide information about the taxonomic ID, the submission number of the strain in a public collection, details of the history of use, and the QPS status, if available. If a GMM is used for the FE production, additional information should be provided according to the Guidance on the Risk Assessment of Genetically Modified Micro-Organisms and their Products Intended for Food and Feed Use [66]. This includes characteristics of the recipient or parental organism, all previous modifications used to create the recipient organism, description techniques used to identify and detect the recipient or parental organism, information on the genetic stability of the recipient organism, information on the origin of the inserted sequences (the donor organism), and characteristics of the used vector.

For all FE obtained from MB sources, except in the case of non-GM QPS strains, the absence of viable cells in the production strain should be investigated by the applicant using a well-described and specific detection method.

Additionally, the presence of DNA from the production strain should also be tested in the product by PCR, targeting a fragment specific to that strain. This applies to all FE obtained from GM production strains and FE obtained from non-GM strains carrying AMR genes. As the threshold, the EFSA statement [67] mentions the following: “The applicant should investigate whether the target DNA is detected in analyses having a detection threshold of 10 ng of DNA per gram or mL of product or lower”. Moreover, in the case of the production strains containing AMR genes, the PCR fragment should not exceed the size of the smallest AMR gene, nor exceed 1 Kb. Currently, the companies themselves are responsible for the quality control of their products and they should verify the compliance with the abovementioned criteria.

The information related to production strains, including genetic modifications, is transmitted confidentially to the EFSA. However, the control laboratories, which would like to control the FE sold on the European market, have no access to this data. Therefore, for GMM’s, no information about the performed modifications, including the presence of AMR genes, or detection methods are available to them. Consequently, this hampers any independent verification by enforcement laboratories for compliance of these FE preparations with the EFSA statement regarding their safety.

Furthermore, the threshold of 10 ng/mL set for risk assessment and the absence of detection methods pose a problem in the context of regulations related to food and feed containing, consisting of, or produced from genetically modified organisms (GMO’s) [68,69] (see Section 6).

## 5. Overview of the Submitted FE Dossiers and Identification of Key Information

In order to obtain an overview of the enzymes produced and used on the European market, an analysis was performed of the Food Enzyme Applications Submitted to the Commission within the Legal Deadline list, published by the EC [28]. This list was constructed following European regulation (EC) No. 1332/2008.

This list of submitted dossiers could be used to obtain an overview of the enzymes produced and commercialized on the European market, as well as their production sources and food applications. A total of 303 dossiers were submitted to the EC within the legal deadline, which was from 11 September 2011 to 11 March 2015 (Table 1). However, since then 8 dossiers have been withdrawn by the applicant and 5 dossiers have not been accepted by the EC, and therefore will not be evaluated. Additionally, 11 new dossiers were submitted after the legal deadline.

At the current time, 74 scientific opinions have been performed by the EFSA and 216 evaluations are ongoing.

Further analysis of the submitted dossiers gave insight into the most produced and most used enzymes, and the most common food applications for which these enzymes are used. Table 2 lists the ten most mentioned enzymes and ten most common food applications, along with their dossier counts. Additionally, for the top enzymes, their enzyme class number is also indicated. Among the top ten FE, mainly the hydrolase group (enzyme class number 3) is represented [11,16]. Regarding the food applications in which these enzymes are used, bakery, dairy, and brewing sectors account for the most submitted dossiers.

**Figure 3:**
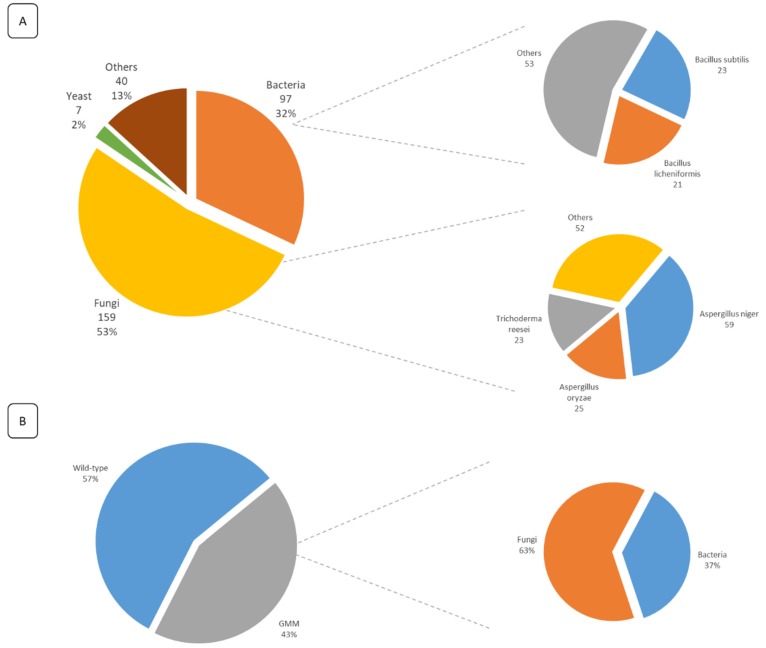
(**A**) Overview of the FE production sources mentioned in all submitted dossiers for safety evaluation (within the legal deadline). For each category, the corresponding number of dossiers is given, along with their percentage according to the total number of submitted dossiers. Additionally, for both bacteria and fungi, more details are given on the most used species, along with the total number of dossiers mentioning their usage. (**B**) The percentage of dossiers mentioning the use of GM strains as the FE production source. For the genetically modified micro-organisms (GMM), more detailed information is given on the percentage of genetically modified (GM) fungal and bacterial strains.

Additionally, the analysis of the submitted FE dossiers allowed the identification of their production sources. It was observed that 87% of the FE are obtained by MB fermentation and 13% through extraction from plants and animals. From those obtained through MB fermentation, over 50% of the FE are produced by fungi, 32% are produced by bacteria, and 2% are produced by yeast. Of the FE obtained by extraction, 47.5% are obtained from animals and 52.5% from plants (Figure 3A). Moreover, 50% of all submitted dossiers are produced by only 5 species, namely *Aspergillus niger*, *A. oryzae*, *Trichoderma reesei*, *Bacillus subtilis*, and *B. licheniformis*. It was also observed that 43% of all submitted dossiers mentioned the use of GM strains for the production of the enzymes (Figure 3B). Of those enzymes obtained from GM strains, 63% are produced by a fungal strain and 37% by a bacterial strain.

Lastly, analysis of the QPS list published by the EFSA regarding the FE-producing MO highlighted that the majority of those species are not granted QPS status [59].

Regarding the QPS status of the FE-producing bacterial strains, 33% of the identified bacterial production species were granted QPS status (Table 3). Of those not recommended for the QPS list, 6 species, namely *Arthrobacter ramosus, Bacillus circulans, Geobacillus pallidus, Geobacillus caldoproteolyticus, Paenibacillus macerans*, and *Pullulanibacillus naganoensis*, would need a complete assessment due to the limited body of knowledge on their safe history of use or presence in food and feed. The *Streptomyces* species produce antibiotics and other secondary metabolites, which may be toxic and are, therefore, not considered for the QPS list. Both *Protaminobacter rubrum* and *Chryseobacterium proteolyticum* are not valid species names, and therefore these taxonomical units cannot be considered for the QPS list. The remaining 5 species, namely *Cellulosimicrobium cellulans, Corynebacterium glutamicum, Escherichia coli, Klebsiella pneumonia*, and *Pseudomonas fluorescens*, have pathogenic potential.

Regarding the filamentous fungi, none of them are granted with the QPS status, mainly due to their ability to produce mycotoxins. All FE-producing yeast species are granted with the QPS status, except *Candida rugose*, which has been described as an “emerging” human fungal pathogen and is well-known for causing mastitis.

However, this does not mean that those bacterial and fungal strains are not recognized as safe (GRAS) and pose a safety problem when used for enzyme production. The consequence is merely that EFSA needs to perform a more in-depth safety and risk assessment than for strains granted with the QPS status.

## 6. Food Enzymes and Related GMO Regulations

### 6.1. Issues Related to the Presence of GMM in the Food Chain

GM food is defined as “food containing, consisting of, or produced from genetically modified organisms (GMO)”, according to the definitions laid down in Article 2 of the directive 2001/18/EC and Recital (16) of (EC) No. 1829/2003 [68,69]. The situation for FE in the context of this regulation is relatively complex. Indeed, “produced from GMOs” means derived in whole or in part from GMO’s, but does not contain or consist of GMO’s. Whether or not a food has been produced “from” or “with” a GMO depends on whether or not material derived from the GM source is present in the food. Food and feed produced “with” a GMO are not covered by the regulation (EC) 1829/2003, and therefore processing aids are not included in the scope of the regulation. This is also clarified in Recital (9) of (EC) 641/2004, which stipulates the implementation of (EC) No. 1829/2003.

All other FE fall under the (EC) No. 1829/2003 if GMM-producing FE are present in commercialized FE preparations. Such GMM would, therefore, need an authorization according to both (EC) No. 1829/2003 and (EC) No. 1332/2008 regulations.

In order to determine whether or not (EC) No. 1829/2003 is applicable to a FE, two steps need to be evaluated. First, it must be determined whether the enzyme is used as a processing aid or as an ingredient. Then, for a processing aid, it must be determined whether or not the use of the enzyme can cause the presence of material derived from the GM source in the produced food product. The regulation (EC) No. 1829/2003 applies if material from the GM source is present in the food product or enzyme preparation.

For all FE falling under regulation (EC) No. 1829/2003 and for which no dossier has been submitted in the context of this regulation, zero tolerance is applied to the GM production strain.

This poses a problem in comparison with the detection threshold of 10 ng of DNA per gram or mL stated by EFSA in the context of the safety evaluation. If this value is converted to genomic copy numbers, this corresponds to an estimation of at least 10^6^ estimated genome copy numbers per gram or mL in the case of *B. subtilis.* The requested performance of the official real-time PCR methods used for GMO control in food and feed products has a limit of detection (LOD) lower than 25 genome copies. Therefore, even if the enzyme is diluted in FE preparation, the difference of magnitude between these 2 thresholds could lead to the detection of unexpected contaminants with GMM, which are used to produce these FE. Moreover, as no FE dossiers have been submitted under regulation (EC) No. 1829/2003, these GMM are automatically considered unauthorized, for which zero tolerance is applied.

### 6.2. Case Study: Unauthorized GMM in A Commercialized Protease Product

#### 6.2.1. Developed Strategy to Detect GMM in Food

**Figure 4:**
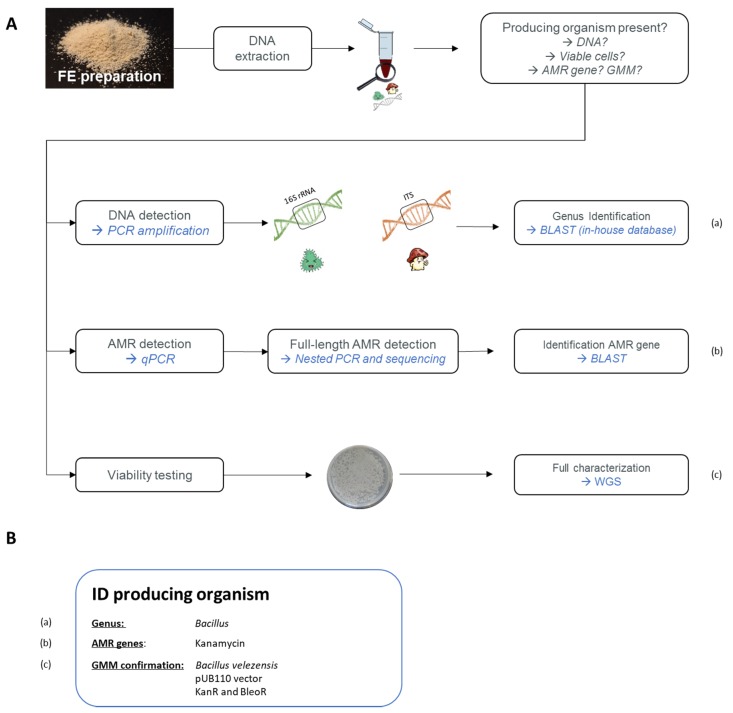
(**A**) General overview of a developed strategy targeting the unexpected presence of FE-producing micro-organisms, with special attention given to GMM strains. (a) First, by targeting the 16S rRNA sequence and the internal transcribed spacer (ITS) region, the potential presence of bacterial and fungal DNA, respectively< can be detected. Identification of an amplicon is obtained by sequencing followed by blasting against in-house databases. (b) The presence of antimicrobial resistance (AMR) genes is targeted using qPCR amplification. If a positive signal is obtained, the full length of the gene is amplified using a nested PCR and then sequenced. The identification of detected AMR genes is verified by blasting. (c) As an additional analysis, a viability assessment is performed. If colonies are obtained, first the two above methods are repeated to confirm the previously obtained results and to select GMM colonies if AMR genes have been detected. A full characterization can consequently be obtained by whole-genome sequencing (WGS) of the obtained colonies. The genetic modifications can be characterized and the identity of the GMM can be demonstrated. (**B**) Using the strategy shown in (**A**), a detected micro-organism can be identified. Here, the example of the GMM-producing protease (RASFF2019.3332) is represented.

Given previous unexpected GMM contaminations in commercialized MB fermentation products, such as in the feed additive vitamin B2 (RASFF2014.1249, RASFF2018.2755, and RASFF2019.3216), competent authorities are highly interested in controlling matrices produced by GMM, such as FE preparations. The performed analyses would target the presence of AMR genes in the context of food and feed safety, along with GMO regulation, which is associated with the freedom of choice of the consumer under EU (EC) No. 1829/2003 [69]. As previously mentioned, information regarding the MB production sources, especially for GMMs, is only communicated confidentially to the EFSA. Therefore, due to the absence of information related to the genetic modifications in GMM, Belgian enforcement laboratories, with the support of the relevant Belgian authorities, have recently developed a strategy to target the unexpected presence of GMM strains (see Figure 4A) [70].

On the one hand, the presence of MB strains was investigated. To this end, a PCR amplification was applied, targeting either the 16S rRNA for bacteria or the internal transcribed spacer (ITS) for fungi, followed by sequencing. The possible observed MB contaminations were then identified down to the genus and species levels using an in-house constructed database, containing 16S and ITS reference sequences, belonging to the MB species used by the food and feed industry to produce FE (Figure 4A, (a)) [71,72].

On the other hand, the potential presence of AMR genes, which are often used as selection markers for GMM, was assessed. Based on a patent analysis, key AMR genes were identified. These are frequently used as selection makers in GM bacterial strains producing MB fermentation products. By combining a *chloramphenicol acetyl-transferase* (*cat*) gene (GenBank: NC_002013.1) conferring CmR, an *aminoglycoside adenyltransferase* (*aadD*) gene (GenBank: M19465.1) conferring KanR, and a tetracycline (*tet-L*) gene (GenBank: D00946.1) conferring TetR, more than 80% of the inventoried patents were covered [72]. On this basis, a strategy targeting these key AMR genes was developed. First, their potential presence was tested by real-time PCR, and in the case of positive qPCR signals, their full-length was determined by conventional PCR followed by sequencing in order to identify potential food safety issues for FE (Figure 4A, (b)) [72,73]. A similar approach would need to be developed to target GM yeasts and fungi.

Using the proposed strategy as a first generic screening, information relating to the potential contamination of FE preparations with MB DNA or with full-length key AMR genes can be obtained for FE (Figure 4B). Moreover, the presence of full-length AMR genes is a strong indication of the potential presence of GMM in FE preparation, meaning that the product would need further investigation.

#### 6.2.2. Application of the Proposed Strategy

As a case study, the proposed strategy was applied on a protease FE preparation, which has been commercialized on the European market (Figure 4B). On this basis, several observations were made. First, the presence of bacterial DNA was detected in the FE preparation, which was identified as *Bacillus* [71]. Additionally, the screening of the product for key AMR genes demonstrated the presence of the full-length *aadD* gene, conferring KanR [73]. Lastly, viable *Bacillus* cells were also isolated from this food enzyme preparation [71]. The isolated cells were consequently analyzed again using the above mentioned PCR and qPCR methods to verify the presence of the full-length AMR genes.

The isolated *Bacillus* strain was then submitted to a whole-genome sequencing (WGS) analysis. This resulted in the identification and characterization of an unauthorized GM *Bacillus velezensis* overproducing protease. This GMM harbored the pUB110 shuttle vector, containing both the KanR and bleomycin resistance (BleoR) genes. With the generated data, real-time PCR methods specific to this GMM were also developed to support enforcement laboratories in the control of FE preparations [74]. These findings resulted in the RASFF2019.3332 notification and the consequent removal of this product from the European market.

### 6.3. Concluding Remarks

In this review, the usefulness of GMM for FE production has been highlighted, mainly due to the possibility to increase the enzyme yield and the safety advantages when an enzyme is originally produced by pathogens.

The case study, as well as other RASFF (Rapid Alert System for Food and Feed) notifications related to the presence of GMM in fermentation products, highlights the importance of analyzing FE preparations and other fermentation products. Additionally, it emphasizes that besides the safety controls performed by the FE industry itself, additional analyses should be performed by independent control laboratories. This control is important in order to verify the compliance of the submitted dossiers to EFSA with all appropriate criteria regarding the absence of viable cells and associated DNA from the production source in FE preparations. However, it also highlights the need for appropriate detection methods that are available to all enforcement laboratories for generic screening or specific detection of GMM, which could be present in FE preparations.

Additionally, an inconsistency was displayed between the limit of detection for GMO detection methods used by GMO enforcement laboratories and the EFSA statement about the absence of the production strain in FE preparations, mentioning a detection threshold of 10 ng of DNA per gram or mL, which is several orders of magnitude higher than the LOD for GMO detection. Therefore, harmonization would be needed at the European level.

Lastly, the case study highlights the issues related to the dissemination of AMR genes due to their potential acquisition by the human commensal flora, via direct food consumption and by environmental bacteria through contact with soil and water surfaces or food waste [75]. This is especially the case as a living GMM was found in a FE preparation [71]. For GM plants, which are used as ingredients in food and feed, the products placed on the market cannot contain AMR genes categorized in group II (i.e., CmR genes) [76]. However, for GM plants, the risk of horizontal gene transfer and consequent dissemination of AMR into the environment is considerably lower, considering that a transfer to MO must occur first. It is not clear why the same precautionary principle is not applied to FE preparations, especially as alternative selection markers can be used to avoid the use of AMR genes.

## Figures and Tables

**Table 1:** Overview of all submitted food enzyme dossiers to the European Commission for evaluation and incorporation in the community list, according to European Commission (EC) No. 1332/2008. Dossiers that were withdrawn, not accepted, or submitted after the legal deadline are also indicated. Lastly, the current status of performed evaluations is given.

Dossier Status	
Submitted dossiers (Within legal deadline)	303
Withdrawn by applicant	8
Not accepted	5
Submitted dossiers (After legal deadline)	11
Evaluated dossiers	74
Ongoing evaluations	216

**Table 2:** Overview of the top ten food enzymes (FE) mentioned in the 303 submitted dossiers for safety. For each enzyme, the number of submitted dossiers is represented and the enzyme class number is given. The ten most mentioned food application uses are also shown, along with the corresponding number of dossiers.

Enzyme	Enzyme Class	Dossier Count	Application of the Enzyme in the Food Industry	Dossier Count
**Alpha-amylase**	3	31	Bakery products and other cereal-based products (e.g., pasta, noodles, snacks)	70
**Triacylglycerol lipase**	3	21	Dairy processing (whey processing)	63
**Xylanase**	3	21	Flavoring production	51
**Beta-galactosidase (lactase)**	3	12	Beer and other cereal-based beverages	49
**Glucoamylase**	3	12	Starch processing	42
**Protease**	3	10	Cereal-based distilled alcoholic beverages	37
**Endo-1,3(4)-β-glucanase**	3	9	Fruit and vegetable processing	36
**Cellulase**	3	8	Protein processing	34
**Cyclomaltodextrin glucanotransferase**	2	7	Yeast processing	32
**Polygalacturonase**	3	7	Processing of oils and fats	18

**Table 3:** Overview of the qualified presumption of safety (QPS) status of the FE-producing micro-organisms (MO) for which a dossier has been submitted to the EFSA for a safety evaluation and subsequent incorporation into the FE community list. The first section contains all bacterial species, the second section contains all filamentous fungal species, and the last section contains the yeast species.

Genus	Species	QPS	Genus	Species	QPS	Genus	Species	QPS
*Arthrobacter*	*ramosus*	No	*Pullulanibacillus*	*naganoensis*	No	*Leptographium*	*procerum*	No
*Bacillus*	*licheniformis*	Yes	*Streptomyces*	*violaceoruber*	No	*Mucor*	*javanicus*	No
*Bacillus*	*subtillis*	Yes	*Streptomyces*	*murinus*	No	*Penicillium*	*roqueforti*	No
*Bacillus*	*circulans*	No	*Streptomyces*	*netropsis*	No	*Penicillium*	*camemberti*	No
*Bacillus*	*pumilus*	Yes	*Streptomyces*	*mobaraensis*	No	*Penicillium*	*multicolor*	No
*Bacillus*	*amyloliquefaciens*	Yes	*Streptomyces*	*rubiginosus*	No	*Penicillium*	*citrinium*	No
*Bacillus*	*flexus*	Yes	*Aspergillus*	*oryzae*	No	*Penicillium*	*decumbens*	No
*Cellulosimicrobium*	*cellulans*	No	*Aspergillus*	*niger*	No	*Penicillium*	*chrysogenum*	No
*Chryseobacterium*	*proteolyticum*	No	*Aspergillus*	*niger* *agg.*	No	*Penicillium*	*funiculosum*	No
*Corynebacterium*	*glutamicum*	No	*Aspergillus*	*niger macrosporus*	No	*Rhizomucor*	*miehei*	No
*Escherichia*	*coli*	No	*Aspergillus*	*niger awamori*	No	*Rhizopus*	*oryzae*	No
*Geobacillus*	*stearothermophilus*	Yes	*Aspergillus*	*fijiensis*	No	*Rhizopus*	*niveus*	No
*Geobacillus*	*pallidus*	No	*Aspergillus*	*acidus*	No	*Talaromyces*	*pinophilus*	No
*Geobacillus*	*caldoproteolyticus*	No	*Aspergillus*	*aculeatus*	No	*Talaromyces*	*emersonii*	No
*Klebsiella*	*pneumoniae*	No	*Aspergillus*	*melleus*	No	*Trametes*	*hirsuta*	No
*Lactobacillus*	*fermentum*	Yes	*Chaetomium*	*gracile*	No	*Trichoderma*	*reesei*	No
*Lactococcus*	*lactis*	Yes	*Chaetomium*	*erraticum*	No	*Trichoderma*	*citrinoviride*	No
*Leuconostoc*	*citreum*	Yes	*Cryphonectria*	*parasitica*	No	*Trichoderma*	*viride*	No
*Microbacterium*	*imperiale*	Yes	*Sporobolomyces*	*singularis*	No			
*Paenibacillus*	*macerans*	No	*Disporotrichum*	*dimorphosporum*	No	*Candida*	*cylindracea*	Yes
*Paenibacillus*	*alginolyticus*	No	*Boletus*	*edulis*	No	*Candida*	*rugosa*	No
*Protaminobacter*	*rubrum*	No	*Fusarium*	*venenatum*	No	*Kluyveromyces*	*lactis*	Yes
*Pseudomonas*	*fluorescens*	No	*Hansenula*	*polymorpha*	No	*Pichia*	*pastori*	Yes
*Pseudomonas*	*amyloderamosa*	No	*Humicola*	*insolens*	No	*Saccharomyces*	*cerevisiae*	Yes

## References

[1] Robinson P.K. (2015). Enzymes: Principles and biotechnological applications. Essays Biochem..

[2] (2016). Industrial Enzymes Market by Type (Amylases, Cellulases, Proteases, Lipases, and Phytases), Application (Food & Beverages, Cleaning Agents, and Animal Feed), Source (Microorganism, Plant, and Animal), and Region—Global Forecast to 2022. Market Research Report. https://www.marketsandmarkets.com/Market-Reports/industrial-enzymes-market-237327836.html.

[3] Chapman J., Ismail A.E., Dinu C.Z. (2018). Industrial applications of enzymes: Recent advances, techniques, and outlooks. Catalysts.

[4] Raveendran S., Parameswaran B., Ummalyma S.B., Abraham A., Mathew A.K., Madhavan A., Rebello S., Pandey A. (2018). Applications of microbial enzymes in food industry. Food Technol. Biotechnol..

[5] Singh R., Kumar M., Mittal A., Kumar P. (2016). Microbial enzymes: Industrial progress in 21st century. 3 Biotech.

[6] Liu X., Kokare C. (2017). Microbial Enzymes of Use in Industry. Biotechnology of Microbial Enzymes.

[7] The European Parliament and the Council of the European Union (2008). Regulation (EC) No 1331/2008 of the European Parliament and of the Council of 16 December 2008 establishing a common authorisation procedure for food additives, food enzymes and food flavourings. Off. J. Eur. Union.

[8] The European Parliament and the Council of the European Union (2008). Regulation (EC) No 1332/2008 of the European Parliament and of the Council of 16 December 2008 on food enzymes and amending Council Directive 83/417/EEC, Council Regulation (EC) No 1493/1999, Directive 2000/13/EC, Council Directive 2001/112/EC and Regulation (EC) No 258/97. Off. J. Eur. Union.

[9] The European Parliament and the Council of the European Union (2008). Regulation (EC) No 1334/2008 of the european parliament and of the council of 16 December 2008 on flavourings and certain food ingredients with flavouring properties for use in and on foods and amending Council Regulation (EEC) No 1601/91, Regulations (EC) No 2232/96 and (EC) No 110/2008 and Directive 2000/13/EC. Off. J. Eur. Union.

[10] Zhang Y., Geary T., Simpson B.K. (2019). Genetically modified food enzymes: A review. Curr. Opin. Food Sci..

[11] Patel A.K., Singhania R.R., Pandey A. (2017). Production, Purification, and Application of Microbial Enzymes.

[12] Sirisha V.L., Jain A., Jain A. (2016). Enzyme Immobilization: An Overview on Methods, Support Material, and Applications of Immobilized Enzymes.

[13] Nedovic V., Kalusevic A., Manojlovic V., Levic S., Bugarski B. (2011). An overview of encapsulation technologies for food applications. Procedia Food Sci..

[14] Fernandes P. (2010). Enzymes in Food Processing: A Condensed Overview on Strategies for Better Biocatalysts. Enzym. Res..

[15] JECFA General Specifications and Considerations for Enzyme Preparations. http://www.fao.org/food/food-safety-quality/scientific-advice/jecfa/jecfa-additives/enzymes/en/.

[16] Fernandes P., Carvalho F. (2017). Microbial Enzymes for the Food Industry.

[17] Saranraj P., Naidu M.A. (2014). Microbial Pectinases: A Review. Glob. J. Tradit. Med. Syst..

[18] Demain A.L., Vaishnav P. (2009). Production of recombinant proteins by microbes and higher organisms. Biotechnol. Adv..

[19] Hjort C. (2007). Industrial Enzyle Production for Food Applications.

[20] Chang M., Chu X., Lv J., Li Q., Tian J., Wu N. (2016). Improving the thermostability of acidic pullulanase from bacillus naganoensis by rational design. PLoS ONE.

[21] Vieille C., Zeikus G.J. (2001). Hyperthermophilic Enzymes. Microbiol. Mol. Biol. Rev..

[22] Olempska-Beer Z.S., Merker R.I., Ditto M.D., DiNovi M.J. (2006). Food-processing enzymes from recombinant microorganisms—A review. Regul. Toxicol. Pharmacol..

[23] Trono D. (2019). Recombinant Enzymes in the Food and Pharmaceutical Industries.

[24] Hui Y.H., Nip W.K., Nollet L.M.L., Paliyath G., Simpson B.K. (2006). Food Biochemistry and Food Processing.

[25] Rieder L., Teuschler N., Ebner K., Glieder A. (2015). Eukaryotic Expressin Systems for Industrial Enzymes. Industrial Enzyme Applications.

[26] Yan S., Wu G. (2017). Bottleneck in secretion of α-amylase in Bacillus subtilis. Microb. Cell Fact..

[27] Meyer V. (2008). Genetic engineering of filamentous fungi—Progress, obstacles and future trends. Biotechnol. Adv..

[28] European Commission (2016). Food Enzyme Applications Submitted to the Commission within the Legal Deadline.

[29] EFSA (2014). Panel on Food Contact Material, Enzymes, Flavourings and Processing Aids (CEF). Scientific Opinion on lipase from a genetically modified strain of Aspergillus oryzae (strain NZYM-AL). EFSA J..

[30] Van Dijck P.W.M., Selten G.C.M., Hempenius R.A. (2003). On the safety of a new generation of DSM Aspergillus niger enzyme production strains. Regul. Toxicol. Pharmacol..

[31] Clyne R.K., Kelly T.J. (1997). Identification of autonomously replicating sequence (ARS) elements in eukaryotic cells. Methods Companion Methods Enzymol..

[32] Vile R. (1992). Selectable markers for eukaryotic cells. Methods Mol. Biol..

[33] Pronk J.T. (2002). Treatments for Osteoarthritis in Pets Continue to Evolve. Appl. Environ. Microbiol..

[34] Lopes T.S., Klootwijk J., Veenstra A.E., van der Aar P.C., van Heerikhuizen H., Raúe H.A., Planta R.J. (1989). High-copy-number integration into the ribosomal DNA of Saccharomyces cerevisiae: A new vector for high-level expression. Gene.

[35] Sunga A.J., Tolstorukov I., Cregg J.M. (2008). Posttransformational vector amplification in the yeast Pichia pastoris. FEMS Yeast Res..

[36] Fitz E., Wanka F., Seiboth B. (2018). The promoter toolbox for recombinant gene expression in trichoderma reesei. Front. Bioeng. Biotechnol..

[37] Christensen T., Woeldike H., Boel E., Mortensen S.B., Hjortshoej K., Thim L., Hansen M.T. (1988). High level expression of recombinant genes in Aspergillus oryzae. Nat. Publ. Gr..

[38] Liu T., Wang T., Li X., Liu X. (2008). Improved heterologous gene expression in Trichoderma reesei by cellobiohydrolase i gene (cbh1) promoter optimization. Acta Biochim. Biophys. Sin..

[39] Zou G., Shi S., Jiang Y., van den Brink J., de Vries R.P., Chen L., Zhang J., Ma L., Wang C., Zhou Z. (2012). Construction of a cellulase hyper-expression system in Trichoderma reesei by promoter and enzyme engineering. Microb. Cell Fact..

[40] Rajamanickam V., Metzger K., Schmid C., Spadiut O. (2017). A novel bi-directional promoter system allows tunable recombinant protein production in Pichia pastoris. Microb. Cell Fact..

[41] Yang S., Sleight S.C., Sauro H.M. (2013). Rationally designed bidirectional promoter improves the evolutionary stability of synthetic genetic circuits. Nucleic Acids Res..

[42] Vogl T., Kickenweiz T., Pitzer J., Sturmberger L., Weninger A., Biggs B.W., Köhler E.M., Baumschlager A., Fischer J.E., Hyden P. (2018). Engineered bidirectional promoters enable rapid multi-gene co-expression optimization. Nat. Commun..

[43] Song R., Zhai Q., Sun L., Huang E., Zhang Y., Zhu Y., Guo Q., Tian Y., Zhao B., Lu H. (2019). CRISPR/Cas9 genome editing technology in filamentous fungi: Progress and perspective. Appl. Microbiol. Biotechnol..

[44] Donohoue P.D., Barrangou R., May A.P. (2018). Advances in Industrial Biotechnology Using CRISPR-Cas Systems. Trends Biotechnol..

[45] Börner R.A., Kandasamy V., Axelsen A.M., Nielsen A.T., Bosma E.F. (2019). Genome editing of lactic acid bacteria: Opportunities for food, feed, pharma and biotech. FEMS Microbiol. Lett..

[46] Zhang K., Su L., Wu J. (2018). Enhanced extracellular pullulanase production in Bacillus subtilis using protease-deficient strains and optimal feeding. Appl. Microbiol. Biotechnol..

[47] Zhang K., Duan X., Wu J. (2016). Multigene disruption in undomesticated Bacillus subtilis ATCC 6051a using the CRISPR/Cas9 system. Sci. Rep..

[48] Salazar-Cerezo S., Kun R.S., de Vries R.P., Garrigues S. (2020). CRISPR/Cas9 technology enables the development of the filamentous ascomycete fungus Penicillium subrubescens as a new industrial enzyme producer. Enzym. Microb. Technol..

[49] Sanchez S., Demain A.L. (2017). Useful Microbial Enzymes—An Introduction.

[50] The European Commission (2011). Commission Regulation (EU) N° 1129/2011 of 11 November 2011 amending Annex II to Regulation (EC) N° 1333/2008 of the European Parliament and of the Council by establishing a Union list of food additives. Off. J. Eur. Union.

[51] Cerutti G., Boudot J., Bournigal J.M., Rousseau L. (2006). Arrêté du 19 Octobre 2006 relatif à L’emploi d’auxIliaires Technologiques Dans la Fabrication de Certaines Denrées Alimentaires. https://www.legifrance.gouv.fr/affichTexte.do?cidTexte=LEGITEXT000020667468#LEGISCTA000020667473.

[52] Fenger A. Bekendtgørelse om tilsætninger mv. til fødevarer. https://www.retsinformation.dk/eli/lta/2018/1247.

[53] Publications Office of the EU (2012). Commission Regulation (EU) No 231/2012 of 9 March 2012 Laying Down Specifications for Food Additives Listed in Annexes II and III to Regulation (EC) No 1333/2008 of the European Parliament and of the Council.

[54] The Council of the European Union (1999). Council regulation (EC) No 1493/1999 of 17 may 1999 on the common organisation of the market in wine. Off. J. Eur. Communities.

[55] The European Commission (2011). Regulation (Eu) No 234/2011 of 10 March 2011 implementing Regulation (EC) No 1331/2008 of the European Parliament and of the Council establishing a common authorisation procedure for food additives, food enzymes and food flavourings. Off. J. Eur. Union.

[56] The European Commission (2012). Commission Implementing. Regulation (EU) No 562/2012 of 27 June 2012 amending Commission Regulation (EU) No 234/2011 with regard to specific data required for risk assessment of food enzymes. Off. J. Eur. Union.

[57] The European Parliament and the Council of the European Union (2000). Directive 2000/13/EC on the approximation of the laws of the Member States relating to the labelling, presentation and advertising of foodstuffs. Off. J. Eur. Union.

[58] Herman L., Chemaly M., Cocconcelli P.S., Fernandez P., Klein G., Peixe L., Prieto M., Querol A., Suarez J.E., Sundh I. (2019). The qualified presumption of safety assessment and its role in EFSA risk evaluations: 15 years past. FEMS Microbiol. Lett..

[59] Ricci A., Allende A., Bolton D., Chemaly M., Davies R., Girones R., Herman L., Koutsoumanis K., Lindqvist R., Nørrung B. (2017). Scientific Opinion on the update of the list of QPS-recommended biological agents intentionally added to food or feed as notified to EFSA. EFSA J..

[60] European Food Safety Authority (2020). Scientific opinion on the update of the list of QPS-recommended biological agents intentionally added to food or feed as notified to EFSA. EFSA J..

[61] Sewalt V., Shanahan D., Gregg L., La Marta J., Carillo R. (2016). The Generally Recognized as Safe (GRAS) process for industrial microbial enzymes. Ind. Biotechnol..

[62] Pariza M.W., Johnson E.A. (2001). Evaluating the safety of microbial enzyme preparations used in food processing: Update for a new century. Regul. Toxicol. Pharmacol..

[63] European Food Safety Authority (2014). Administrative Guidance to applicants on the suitability check of applications for authorisation of food enzymes submitted under Regulation (EC) No 1332/2008. EFSA Support. Publ..

[64] European Food Safety Authority (2014). Explanatory Note for the Guidance of the Scientific Panel of Food Contact Materials, Enzymes, Flavourings and Processing Aids (CEF) on the Submission of a Dossier on Food Enzymes. EFSA Support. Publ..

[65] Anadón A., Bell D., Binderup M.L., Bursch W., Castle L., Crebelli R., Engel K.H., Franz R., Gontard N., Haertlé T. (2009). Guidance of the Scientific Panel of Food Contact Materials, Enzymes, Flavourings and Processing Aids (CEF) on the Submission of a Dossier on Food Enzymes for Safety Evaluation by the Scientific Panel of Food Contact Material, Enzymes, Flavourings and Proc. EFSA J..

[66] EFSA, Panel on Genetically Modified Organisms (GMO) (2011). Guidance on the risk assessment of genetically modified microorganisms and their products intended for food and feed use. EFSA J..

[67] Silano V., Baviera J.M.B., Bolognesi C., Brüschweiler B.J., Cocconcelli P.S., Crebelli R., Gott D.M., Grob K., Lampi E., Mortensen A. (2019). Characterisation of microorganisms used for the production of food enzymes. EFSA J..

[68] The European Parliament and the Council of the European Union (2001). The new directive 2001/18/EC on the deliberate release of genetically modified organisms into the environment: Changes and perspectives. Off. J. Eur. Communities.

[69] The European Parliament and the Council of the European Union (2003). Regulation (EC) No 1829/2003 of the European Parliament and of the council on genetically modified food and feed. Off. J. Eur. Union.

[70] FOD Volksgezondheid (2017). Contractueel Onderzoek.

[71] Deckers M., Vanneste K., Winand F., DeKeersmaecker S.C.J., Denayer S., Heyndrickx M., Deforce D., Fraiture M.A., Roosens N.H.C. (2020). Strategy for the identification of micro-organisms producing food and feed products: Bacteria producing food enzymes as study case. Food Chem..

[72] Fraiture M.A., Deckers M., Papazova N., Roosens N.H.C. (2020). Detection strategy targeting a chloramphenicol resistance gene from genetically modified bacteria in food and feed products. Food Control.

[73] Fraiture M., Deckers M., Papazova N., Roosens N.H.C. (2020). Strategy to control the presence of antimicrobial resistance genes in food and feed bacterial fermentation products. Int. J. Food Microbiol..

[74] Fraiture M. (2020). Next-generation sequencing: A key tool to identify unauthorized genetically modified microorganisms in food enzyme preparations. Sci. Rep..

[75] Likotrafiti E., Oniciuc E., Prieto M., Santos J., Lopez S., Alvarez-Ordonez A. (2018). Risk assessment of antimicrobial resistance along the food chain through culture-independent methodologies. EFSA J..

[76] EFSA (2004). Opinion of the Scientific Panel on Genetically Modified Organisms on the use of antibiotic resistance genes as marker genes in genetically modified plants. EFSA J..

